# From Feasibility to Individualization: Surgery for Breast Cancer Liver and Lung Metastases

**DOI:** 10.3390/cancers18050822

**Published:** 2026-03-03

**Authors:** Martina Greco, Calogero Cipolla, Chiara Mesi, Alessio Ciminna, Daniela Sambataro, Giuseppa Scandurra, Simona Lupo, Gaspare Cannata, Luca Giacomelli, Vittorio Gebbia, Maria Rosaria Valerio

**Affiliations:** 1 UOC Medical Oncology, AOUP Paolo Giaccone Palermo, 90127 Palermo, Italy; doc.martinagreco@gmail.com (M.G.); chiaramesi95@gmail.com (C.M.); alessio.ciminna@libero.it (A.C.); 2 Breast Unit, AOUP Paolo Giaccone Palermo, 90127 Palermo, Italy; calogero.cipolla@unipa.it (C.C.); simona.lupo@policlinico.pa.it (S.L.); gaspare.cannata@policlinico.pa.it (G.C.); 3 Department of Biomedicina, Neuroscienze e Diagnostica Avanzata, University of Palermo, 90127 Palermo, Italy; 4 Medical Oncology, Department of Medicine and Surgery, Kore University of Enna, 94100 Enna, Italy; daniela.sambataro@unikore.it (D.S.); giuseppa.scandurra@unikore.it (G.S.); 5 Medical Oncology Unit, Ospedale Umberto I, 94100 Enna, Italy; mariarosaria.valerio@unipa.it; 6 Medical Oncology Unit, Ospedale Cannizzaro, 95021 Catania, Italy; 7 Polistudium SRL, 20121 Milan, Italy; luca.giacomelli@polistudium.it; 8 Medical Oncology Unit, CDC Torina, 90145 Palermo, Italy; 9 Department of Surgical Oncological and Oral Sciences, University of Palermo, 90127 Palermo, Italy

**Keywords:** ablation, artificial intelligence, breast cancer, ctDNA, liver metastases, lung metastases, metastasectomy

## Abstract

Surgical resection of liver and lung metastases in breast cancer is increasingly considered a viable option for select patients with oligometastatic disease. Historically regarded as palliative, surgery is now supported by retrospective data suggesting potential survival benefits, particularly in patients with hormone receptor-positive or HER2-positive tumors, long disease-free intervals, and limited metastatic burden. This narrative review summarizes recent evidence on the surgical management of breast cancer metastases to the liver and lung, with a focus on patient selection, perioperative outcomes, and long-term survival. Liver metastasectomy has shown 5-year overall survival rates of up to 60% in well-selected patients, while pulmonary metastasectomy is associated with comparable outcomes when resection is complete and nodal involvement is absent. Minimally invasive techniques and non-surgical approaches, such as microwave ablation and stereotactic radiotherapy, expand treatment options for patients unfit for surgery. The review also explores emerging tools influencing surgical decision-making, including circulating tumor DNA for minimal residual disease detection, transcriptomic profiling to predict organotropism, and artificial intelligence (AI)-driven platforms that assist with surgical planning and multidisciplinary case evaluation. While prospective validation remains limited, these technologies may help redefine surgical candidacy through biologically informed algorithms. Ultimately, the integration of surgery within a multimodal, personalized treatment strategy—guided by systemic control, tumor biology, and evolving digital tools—represents an evolving and biologically informed direction for rigorously selected patients with visceral breast cancer metastases.

## Simple Summary

Breast cancer can spread to distant organs, such as the liver and lungs. Traditionally, when this happens, treatment has focused mainly on medications rather than surgery, because metastatic disease has been considered systemic and incurable. However, in selected patients with a limited number of metastases, surgical removal of these lesions is increasingly being explored as part of a personalized treatment strategy. This review summarizes current evidence on surgery for liver and lung metastases from breast cancer, discussing which patients may benefit, how outcomes compare with non-surgical approaches, and how modern tools such as molecular testing and artificial intelligence may improve decision-making. While promising results have been reported in carefully selected patients, strong randomized evidence is still lacking. Future research is needed to clarify when surgery meaningfully improves long-term outcomes.

## 1. Introduction

Breast cancer (BC) remains the most diagnosed malignancy among women and is a leading cause of cancer-related mortality globally [1]. Despite advancements in early detection and systemic therapies—including endocrine treatment, HER2-targeted agents, and immunotherapy—a significant proportion of patients, estimated at 20–30%, will eventually develop distant metastases [2,3].

The most frequently involved metastatic sites are bone, liver, and lung, with visceral metastases generally portending a poorer prognosis than bone-only disease [1,4]. Liver and lung metastases represent the most common visceral sites of disease spread in metastatic breast cancer (MBC), observed in approximately 15–25% and 10–20% of cases, respectively [5,6]. The prognosis of patients with these metastases varies depending on tumor subtype, metastatic burden, response to systemic treatment, and disease-free interval. Reported median overall survival (OS) for patients with liver metastases from BC ranges from 27 to 74 months, with 5-year survival rates up to 53% in selected surgical series [3,6,7]. For lung metastases, median OS ranges from 20 to 97 months, with 5-year survival reported between 36% and 46% [3,4].

Metastatic dissemination in breast cancer is a biologically heterogeneous process driven by complex interactions between tumor-intrinsic molecular alterations and organ-specific microenvironments. Distinct molecular subtypes exhibit preferential patterns of organotropism, with HER2-positive and triple-negative tumors more frequently associated with visceral spread, including liver and lung involvement. Key pathways implicated in metastatic progression include PI3K/AKT/mTOR signaling, HER2 amplification, *ESR1* mutations, and alterations in cell adhesion and immune evasion mechanisms. These biological features are not only prognostic but also therapeutically actionable. The availability of targeted agents, including CDK4/6 inhibitors, HER2-directed therapies, PI3K inhibitors, PARP inhibitors and antibody–drug conjugates, has significantly improved systemic disease control, thereby expanding the window of opportunity for metastasis-directed local therapies in selected patients. Consequently, understanding the molecular drivers of metastatic behavior is increasingly relevant to surgical decision-making within a multidisciplinary and precision oncology framework.

In recent years, the concept of oligometastatic disease—defined as a state of limited metastatic burden, often involving five or fewer lesions—has reshaped clinical decision-making in MBC [3,8]. In this context, surgical management of metastases, once considered purely palliative, is now being investigated as a potentially life-prolonging strategy in carefully selected patients. Surgical resection of liver or lung metastases may offer survival benefit, particularly when performed in combination with effective systemic therapies and in the absence of widespread disease [3,5,9].

However, evidence supporting metastasectomy in MBC remains largely retrospective and non-randomized, and thus subject to selection bias. Most data derive from observational cohorts or institutional case series, making it difficult to draw definitive conclusions. Nonetheless, several reports have shown encouraging long-term outcomes after resection of liver or lung metastases, especially in patients with hormone receptor-positive or HER2-positive tumors and long disease-free intervals [6,7,10]. A recent meta-analysis involving over 1700 patients confirmed significantly lower mortality at 1, 3, and 5 years in surgically treated patients compared to those receiving systemic therapy alone [11]. A nationwide Swedish case–control study reported a median survival of 77 months after liver resection compared to 28 months in patients treated with systemic therapy alone, reinforcing the potential benefit of surgery in well-selected cases [12].

It is also noteworthy that the biological behavior of metastases may differ by histological subtype. Invasive lobular carcinoma, for instance, is more prone to late and atypical metastatic spread, including to the gastrointestinal tract, which may complicate diagnosis and treatment [13,14]. Personalized and precision medicine approaches are increasingly central to surgical decision-making in metastatic breast cancer. In this context, precision oncology refers to the integration of molecular subtype, genomic alterations, tumor biology, systemic therapy responsiveness, disease kinetics and patient-specific clinical factors into individualized treatment strategies. Rather than relying solely on anatomical resectability or metastatic burden, contemporary surgical evaluation incorporates biological behavior, such as hormone receptor status, HER2 amplification, patterns of organotropism and dynamic response to targeted systemic therapy, to identify patients most likely to benefit from metastasis-directed interventions. This paradigm shift reflects the recognition that metastatic breast cancer is not a uniform entity but a biologically heterogeneous disease requiring tailored therapeutic sequencing within a multidisciplinary framework. Accordingly, surgical intervention should be considered as one component of a personalized treatment algorithm rather than as an isolated technical procedure.

Given the frequency and prognostic relevance of liver and lung metastases in MBC, this review aims to provide a critical evaluation of the surgical management of these two metastatic sites. We will focus on recent evidence, discuss the criteria for patient selection, outline surgical outcomes, and consider emerging minimally invasive and ablative strategies. While metastases to bone or brain are clinically important, our review will center on the liver and lung due to their frequency and the growing clinical interest in their local management [3,15]. Given the predominance of retrospective evidence and the absence of definitive randomized data, interpretation of survival outcomes requires careful consideration of selection bias and evolving systemic therapy landscapes.

## 2. Methods

This article presents a narrative review focusing on the surgical management of liver and lung metastases in patients with breast cancer. The aim was to synthesize and critically appraise recent literature regarding indications, patient selection criteria, surgical outcomes, and the integration of local therapies with systemic treatment in the context of oligometastatic disease. The objective was to provide a clinically oriented and interpretative overview of the available evidence rather than to perform a formal systematic review or meta-analysis.

The literature search was conducted in December 2025 and focused on studies published in English. Databases consulted included PubMed/MEDLINE and Scopus. Search terms included combinations of “breast cancer,” “metastases,” “liver metastases,” “lung metastases,” “metastasectomy,” “surgical resection,” “oligometastatic,” and “local therapy.” For example, the combination of the terms “breast cancer” and “metastasectomy” in PubMed yields approximately 200 publications, reflecting the breadth and heterogeneity of the available literature. Reference lists of relevant articles were also screened to identify additional studies.

Priority was given to studies published in the past 10 years, with particular emphasis on recent work from 2020 onward. Both retrospective and prospective studies were considered, including observational cohorts, registry data, case–control studies, randomized trials where available, as well as expert consensus guidelines and meta-analyses. Studies addressing novel or adjunctive local therapies (e.g., ablation, stereotactic body radiotherapy) were included when relevant to the surgical context. Articles were excluded if they did not provide specific data on surgical outcomes, focused solely on non-visceral metastases, or lacked immediate clinical applicability. Within this non-systematic framework, studies were prioritized based on clinical relevance to liver and lung metastases, methodological robustness, sample size, recency, and the availability of survival outcomes applicable to contemporary systemic therapy eras.

This is not a systematic review, and no formal quality assessment or statistical synthesis of data was performed. Instead, the selected references reflect clinically relevant and methodologically robust contributions to the evolving field of metastasis-directed therapy in breast cancer. Accordingly, no PRISMA-based selection process or structured quality scoring system was applied, consistent with the narrative nature and interpretative aim of the review.

## 3. Liver Metastases

### 3.1. Epidemiology and Clinical Significance

Liver metastases occur in approximately 5–25% of patients with advanced breast cancer and are the third most common site of distant spread after bone and lung [16,17]. While some patients present with liver involvement at diagnosis, others develop metachronous metastases months or years after initial treatment [18]. The prognosis associated with breast cancer liver metastases (BCLM) remains poor, particularly when extrahepatic disease is present, and median OS is frequently reported in the range of 13–30 months [16,17,18]. Compared to bone or lung metastases, liver involvement is associated with a more aggressive clinical course, often reflecting underlying unfavorable tumor biology such as HER2-negative or triple-negative subtypes [17].

### 3.2. Biological and Molecular Characteristics

The receptor profile of liver metastases frequently differs from that of the primary tumor. In a population-based analysis, discordance in estrogen receptor (ER), progesterone receptor (PgR), and HER2 status between primary tumors and liver metastases was observed in 17%, 33%, and 10% of cases, respectively [16]. Subtype conversion occurred in 21% of patients, often resulting in a more aggressive phenotype. Importantly, HER2 amplification in BCLM was associated with improved survival (hazard ratio [HR] 0.28), underscoring the relevance of re-biopsy and molecular reassessment in guiding therapy [16].

Beyond biological subtype, other factors associated with survival in BCLM include patient age, number of liver lesions, and disease-free interval [17,18]. A nomogram based on SEER data for T1–2N0–1 breast cancer patients, with liver metastases identified ER/PR/HER2 status, surgery, and systemic treatment as independent prognostic variables, with a c-index of 0.72 [19].

### 3.3. Surgical Resection of Liver Metastases

Surgical resection of BCLM has historically been considered controversial due to the systemic nature of the disease. However, accumulating retrospective evidence suggests that, in selected patients, hepatic resection may be associated with a significant survival benefit (Table 1). The largest series and meta-analyses to date report median OS ranging from 32 to 60 months following resection, with 5-year survival rates of 25–60%, particularly in hormone receptor-positive or HER2-positive subtypes [18,20].

The European LIBREAST study, one of the largest multicenter registries of patients undergoing liver resection for BCLM, demonstrated a 5-year OS of 60% and a 5-year disease-free survival (DFS) of 29%, though in highly specialized centers [20]. Similarly, data from Masuda et al. confirm prolonged survival in patients with isolated liver metastases treated surgically, particularly when resection margins were negative (R0) and systemic therapy achieved disease control [21]. In these series, liver resection was often part of a multidisciplinary strategy that included chemotherapy, targeted therapy, and endocrine treatment.

Optimal candidates for surgery typically exhibit a limited number of liver lesions (≤3–5), absence of uncontrolled extrahepatic disease, good performance status, and a long disease-free interval from primary diagnosis [18,21]. Hormone receptor positivity, HER2 amplification, and response to systemic therapy prior to resection are also associated with improved outcomes.

Despite the absence of randomized trials, these consistent findings across large observational cohorts suggest a potential role for hepatic surgery in highly selected patients with oligometastatic BCLM. Importantly, surgical decisions must be individualized and made within the context of multidisciplinary team discussions [20].

### 3.4. Local Ablative Therapies

For patients with unresectable BCLM or those unfit for surgery, image-guided local ablative therapies have emerged as viable alternatives. Techniques, such as microwave ablation (MWA), laser interstitial thermal therapy (LITT) and transarterial chemoembolization (TACE), have been employed either alone or in multimodal regimens. These approaches are particularly relevant in the oligometastatic setting, where local control may contribute to prolonged survival and delay systemic progression [18,22,23].

A large single-center retrospective study evaluated over 1100 patients treated with various thermal ablation modalities over a 26-year period [22]. MWA yielded the most favorable outcomes, with a median OS of 5.6 years and 5-year survival rate of 89% in a small but highly selected cohort (*n* = 17). LITT, performed in an earlier era, was associated with a median OS of 2.2 years and a 5-year survival rate of 22%, while combination therapy with LITT and TACE yielded comparable survival (median OS 2.1 years, 5-year survival 15%). TACE alone was associated with poor outcomes (median OS 0.8 years; 5-year survival 4%), likely reflecting treatment in more advanced cases (Table 2).

MWA has several technical advantages over older methods such as radiofrequency ablation, including faster tissue heating, larger ablation volumes, and less susceptibility to heat-sink effects. LITT, while more time-intensive, offers precise thermal monitoring via MRI guidance and remains suitable for lesions ≤ 5 cm in diameter [22].

TACE continues to serve as both a palliative modality and a downstaging tool in combination protocols. When used before ablation, it may reduce tumor vascularity and enhance necrosis [22].

Overall, local ablation offers a valuable therapeutic option for well-selected patients and should be considered as part of a multimodal treatment strategy in the absence of surgical indications. SBRT has also emerged as a non-invasive alternative to surgery in selected patients, with favorable local control and toxicity profiles. Breast cancer histology appears to be associated with better outcomes compared to other primaries [24]. Recent data from the OligoCare prospective cohort confirmed a low incidence of acute grade ≥ 3 toxicities (<1%), reinforcing the safety of SBRT in oligometastatic breast cancer [25]. Recent international consensus recommendations highlight the importance of stratifying future OMBC trials by imaging modality, metastatic site, and biological subtype, and emphasize the integration of modern diagnostics into trial and clinical frameworks [26].

### 3.5. Minimally Invasive vs. Surgical Approaches

The decision between surgical resection and minimally invasive locoregional therapies for BCLM hinges on tumor burden, anatomic resectability, patient comorbidities, and response to systemic therapy. While hepatic resection remains the standard of care in operable oligometastatic disease, image-guided ablative modalities offer less invasive alternatives for patients who are medically inoperable or have unfavorable lesion locations.

MWA and LITT have demonstrated encouraging long-term outcomes in small cohorts. MWA achieved a 5-year survival rate of 89% in a highly selected population, comparable to surgical outcomes in similar patients [22]. Ablative approaches also carry lower perioperative morbidity, shorter hospital stays, and the potential for repeat treatments, making them attractive in frail or elderly populations [22].

However, some surgical series report longer disease-free intervals when complete (R0) resection is feasible, although such comparisons are confounded by differences in patient selection and disease biology. For example, the LIBREAST registry reported a 5-year OS of 60% after hepatic resection, highlighting the potential curative intent in select cases [20]. Conversely, TACE as a monotherapy yields inferior outcomes and is generally reserved for patients with multifocal disease or contraindications to thermal ablation or surgery [22].

In practice, the choice between resection and minimally invasive therapy should be individualized and based on multidisciplinary evaluation. Some centers are adopting combined strategies—using TACE or ablation as neoadjuvant tools to downstage lesions prior to resection or to treat bilobar disease with curative intent [18,22].

The comparative effectiveness of these approaches remains inadequately addressed in prospective studies, underscoring the need for ongoing clinical trials and standardized selection criteria.

### 3.6. Emerging Strategies

The management of BCLM is increasingly guided by individualized, multidisciplinary strategies. A key area of development is conversion therapy, wherein patients with initially unresectable disease are downstaged using systemic therapy or locoregional interventions—such as TACE or thermal ablation—to enable curative-intent surgical resection or combined local therapy [18,22].

Risk-adapted treatment algorithms are also gaining relevance. A recent nomogram, derived from a SEER-based cohort of patients with T1–2N0–1 breast cancer and liver metastases, incorporated clinical and molecular features, such as ER, PR and HER2 status, along with brain and bone metastases, to estimate individualized survival probabilities. This model achieved a concordance index of 0.72 and effectively stratified patients into high- and low-risk groups, offering potential guidance for therapeutic decision-making [19].

Receptor discordance between the primary tumor and liver metastases, including loss of hormone receptor expression or gain of HER2 amplification, has been documented in up to one-third of cases. This highlights the importance of repeat biopsy and molecular re-evaluation at the time of metastatic progression, particularly when local treatment is being considered [16].

The integration of surgery, systemic therapy, and locoregional techniques remains largely based on retrospective evidence. Prospective studies and randomized trials comparing surgical resection with ablative or non-invasive strategies are urgently needed. In the interim, real-world data and multicenter registries may help refine patient selection and clarify the comparative benefits of available interventions.

As the therapeutic landscape continues to evolve, optimal management of BCLM will depend on the coordinated application of biological, anatomical, and clinical factors within a multidisciplinary framework.

## 4. Lung Metastases

### 4.1. Epidemiology and Prognostic Impact

Lung metastases are a frequent manifestation of advanced breast cancer, occurring in up to 20–40% of patients with distant recurrence [27,28]. Isolated pulmonary metastases are less common but represent a distinct clinical scenario that may be amenable to local therapy. The lung is often involved early in metastatic dissemination, particularly in hormone receptor-negative and HER2-positive subtypes [27].

The prognosis of patients with lung metastases varies significantly depending on disease burden, molecular subtype, and the feasibility of local control [29]. Historical data suggest that median OS for patients with metastatic breast cancer involving the lung ranges from 22 to 36 months [28]. In a retrospective study of patients undergoing MWA for breast cancer lung metastases, the reported median OS was 36 months, with 1-, 3- and 5-year OS rates of 96.9%, 53.3% and 17.8%, respectively [28].

Compared to liver or bone metastases, lung involvement may carry a slightly more favorable prognosis in selected patients, particularly when the disease is limited in number and confined to the lung. However, it also remains a marker of systemic progression, and long-term disease control is uncommon in the absence of systemic therapy.

### 4.2. Patient Selection for Pulmonary Metastasectomy

The selection of candidates for pulmonary metastasectomy (PM) requires careful clinical judgment and should be guided by multidisciplinary evaluation. Established criteria include the control of the primary tumor, absence of extrapulmonary metastases (or stable extra-thoracic disease), a limited number of lung lesions (typically ≤ 3), resectability with negative margins, and sufficient cardiopulmonary reserve [30].

A disease-free interval greater than 12–24 months has consistently been associated with better outcomes after PM [30]. Biological subtype also plays a role, with hormone receptor-positive and HER2-positive tumors more likely to benefit from metastasis-directed interventions.

### 4.3. Surgical Techniques and Outcomes

Surgical resection is widely regarded as the standard local approach for isolated or oligometastatic pulmonary metastases from breast cancer in appropriately selected patients (Table 3). The primary objective is to achieve complete resection of all visible disease with negative margins (R0). The most employed procedures are non-anatomical wedge resections, which offer effective tumor removal while preserving lung parenchyma. Segmentectomy or lobectomy may be necessary for centrally located lesions or when margin-negative resection is not otherwise feasible [30].

Minimally invasive surgery, particularly video-assisted thoracoscopic surgery (VATS), has become the preferred approach in most centers due to its favorable perioperative profile. Compared with open thoracotomy, VATS is associated with shorter hospital stays, reduced postoperative pain, faster recovery, and comparable oncologic outcomes in terms of recurrence and survival [30]. The low morbidity and mortality rates of pulmonary metastasectomy are well established: in a multicenter retrospective study, the 30-day mortality rate was 1.1%, and the overall complication rate was approximately 11% [30].

Long-term survival outcomes following metastasectomy are encouraging in selected patients. The 5-year OS rates range from 35% to 60%, depending on factors such as disease-free interval, tumor biology, and extent of disease. Recurrence within the lung occurs in approximately 20–30% of cases, most often after a prolonged disease-free period. However, recurrence does not preclude consideration of repeat surgical intervention, which may offer continued disease control and survival benefit [30].

Surgical resection of lung metastases is therefore a viable and generally safe option, provided that patient selection is rigorous and performed within a multidisciplinary context.

### 4.4. Lymph Node Involvement and Prognostic Significance

The presence of mediastinal or hilar LN metastases at the time of PM is an established negative prognostic factor in patients with breast cancer. Nodal involvement reflects a higher systemic disease burden and more aggressive tumor biology, and it is associated with significantly reduced survival outcomes compared to node-negative patients [30].

In one of the largest retrospective analyses of PM for breast cancer, patients with positive mediastinal or hilar lymph nodes had markedly worse outcomes, with 3-year OS of 38% compared to 69% in node-negative patients [30]. These findings support the recommendation for routine intraoperative lymph node assessment during PM. In the absence of preoperative evidence of nodal disease, systematic sampling or dissection may still be warranted to accurately stage the disease and refine postoperative management.

The decision to proceed with PM should incorporate the likelihood of nodal involvement, particularly in patients with high-risk subtypes or short disease-free intervals. Preoperative imaging, including PET-CT, may assist in identifying suspicious nodes, although sensitivity remains suboptimal. In cases with confirmed or strongly suspected LN metastasis, the role of surgery must be carefully weighed against the potential benefit of systemic therapy or other non-surgical strategies.

Despite its prognostic impact, nodal positivity does not represent an absolute contraindication to PM, especially in cases of controlled disease and favorable performance status. However, outcomes are clearly superior when nodal involvement is absent, reinforcing the need for thorough staging and multidisciplinary decision-making [30].

### 4.5. Repeat Metastasectomy and Long-Term Disease Control

Recurrence following PM is common in breast cancer, occurring in up to 30% of patients within the lungs, often after prolonged disease-free intervals. Importantly, recurrence does not necessarily preclude the possibility of additional surgical intervention. In selected patients, repeat metastasectomy has been shown to be both feasible and beneficial, contributing to extended survival and sustained disease control [30].

The decision to perform a second or even third PM depends on multiple factors, including the location and number of recurrent lesions, patient performance status, interval since prior surgery, and response to systemic therapy. Studies have shown that the morbidity of repeat PM is comparable to that of the initial resection, particularly when using minimally invasive techniques, such as VATS [30].

Although data are limited, survival outcomes following repeat PM appear similar to those observed after the initial surgery in well-selected patients. This suggests that repeated local treatment may be a reasonable strategy within the broader framework of multidisciplinary care, especially in oligo-recurrent disease with slow progression and good systemic control.

Ultimately, the appropriateness of repeat PM must be assessed case by case, with careful attention to evolving disease biology and treatment goals. When pursued, repeat surgical resection has been reported to provide additional disease control in selected cases, although evidence remains limited.

### 4.6. Non-Surgical Ablative Therapies

For patients who are not suitable candidates for pulmonary metastasectomy due to comorbidities, multifocal pulmonary involvement, poor functional status, or surgically inaccessible lesions, non-surgical local treatments have become important tools in the management of breast cancer lung metastases. Image-guided ablative therapies and stereotactic body radiotherapy (SBRT) are increasingly used to achieve local control in oligometastatic or oligoprogressive disease settings.

MWA is among the most widely studied thermal ablation techniques. It offers several advantages, including short procedure times, larger and more uniform ablation zones, and reduced heat-sink effects compared to radiofrequency ablation. In a retrospective study by Meng et al., 32 patients with breast cancer lung metastases underwent CT-guided percutaneous MWA [28]. The technical success rate was 100%, with a primary local control rate of 97.8%. Local progression occurred in 10.9% of lesions, with a median progression time of 10 months. Median OS was 36 months, with 1-, 3- and 5-year OS rates of 96.9%, 53.3%, and 17.8%, respectively. Complications included pneumothorax (23.9%) and pleural effusion (4.3%), but no treatment-related mortality was reported [28].

SBRT is another non-invasive option with excellent local control and favorable safety. In a prospective phase II study, SBRT delivered to lung and liver oligometastases from breast cancer resulted in 1-, 2- and 3-year local control rates of 94.9%, 91%, and 87.5%, respectively. The 3-year OS was 51.9%, despite many patients having extra-thoracic but stable disease [31].

These minimally invasive modalities are particularly valuable in the multidisciplinary management of patients with limited pulmonary disease who are not surgical candidates, offering durable local control with low morbidity. However, the randomized phase II NRG-BR002 trial did not show a survival advantage for adding SBRT or surgery to systemic therapy in newly diagnosed oligometastatic breast cancer, highlighting the need for better biological selection criteria [32]. This need is further supported by recent interim data from the EORTC-ESTRO OligoCare study, which has shown considerable heterogeneity in SBRT dose and fractionation choices, influenced by both primary tumor histology and metastatic site, underscoring the need for harmonized clinical practice [33].

### 4.7. Integration with Systemic Therapy and Multidisciplinary Planning

Effective management of pulmonary metastases in breast cancer relies not only on local control strategies but also on the integration of systemic therapy within a multidisciplinary treatment plan. Surgery or ablation alone is rarely sufficient, as breast cancer metastases reflect underlying systemic disease. Thus, local interventions must be timed and selected based on systemic control, tumor biology and patient-specific factors.

Systemic therapy remains the cornerstone of metastatic breast cancer treatment and is typically administered before or after local interventions. Hormone receptor-positive and HER2-positive subtypes particularly benefit from targeted systemic regimens, which can stabilize or reduce disease burden and facilitate subsequent local therapy. In patients with triple-negative disease, systemic therapy plays a more limited role in long-term disease control, further highlighting the importance of patient selection for aggressive local treatments [30].

The decision to proceed with pulmonary metastasectomy or non-surgical ablation should be made within the context of a MDT, including thoracic surgeons, medical oncologists, radiation oncologists, and radiologists. The MDT should consider disease distribution, treatment response, patient comorbidities, and preferences. Studies have shown that MDT evaluation improves treatment outcomes and optimizes resource allocation [30].

Ultimately, the management of lung metastases in breast cancer must be individualized. Whether through surgery, ablation, or SBRT, local therapies offer a survival advantage only when integrated into a broader therapeutic strategy that accounts for systemic disease dynamics and patient goals.

## 5. Emerging Technologies and Personalized Strategies

The technologies discussed in this section span different stages of clinical development. While some tools, such as circulating tumor DNA (ctDNA) assays for minimal residual disease detection, have demonstrated clinical validity in defined breast cancer settings, their application to metastasis-directed surgical decision-making remains under investigation. Other approaches, including transcriptomic prediction models and artificial intelligence–based surgical planning platforms, are largely exploratory and should currently be considered hypothesis-generating. The following discussion aims to differentiate between validated applications and emerging strategies that require prospective confirmation.

### 5.1. Molecular and Genomic Profiling for Surgical Selection

Recent advances in molecular diagnostics have introduced new opportunities to refine surgical decision-making in metastatic breast cancer, particularly in patients with oligometastatic liver or lung involvement. Molecular profiling—both tissue-based and liquid biopsy—can provide insights into tumor biology, metastatic potential, and minimal residual disease (MRD), thereby informing the appropriateness and timing of surgical interventions.

A key development in this area is the use of ctDNA to detect MRD. In a recent study, Garcia-Murillas et al. demonstrated that personalized ctDNA assays can detect relapse in early-stage breast cancer patients with high specificity and sensitivity, often months before radiologic evidence of progression [34]. Among 141 patients, ctDNA positivity was associated with significantly shorter distant recurrence-free survival. Importantly, in the subset of patients with isolated distant relapse—many of whom had lung or liver involvement—ctDNA positivity preceded clinical recurrence by a median of 11 months. This aligns with the findings of a systematic review and meta-analysis by Cullinane et al., which confirmed that detectable ctDNA in early-stage and advanced breast cancer is significantly associated with shorter disease-free survival and worse outcomes [35]. Recent prospective findings from the c-TRAK TN trial indicate that ctDNA-based intervention may have limited benefit if metastases are already established at the time of detection, highlighting the need for earlier and more sensitive molecular surveillance in high-risk settings [36]. These findings support the clinical validity of ctDNA monitoring for relapse detection; however, its role in selecting candidates for curative-intent local therapies, including surgery, remains investigational and requires prospective validation. Recent translational studies have also emphasized ctDNA’s role in real-time treatment adaptation, resistance detection, and guiding metastasis-directed therapies through liquid biopsy-integrated platforms [37].

In parallel, transcriptomic profiling is shedding light on the molecular drivers of organ-specific metastasis. Cai et al. applied a deep learning approach to identify gene signatures associated with breast cancer metastases to lung and liver, generating predictive models with improved accuracy over traditional statistical methods [38]. These gene sets may eventually contribute to risk stratification and surveillance strategies; however, their integration into surgical decision-making algorithms remains exploratory and has not yet been validated in prospective clinical trials. Recent transcriptomic analyses by Zhang et al. have further delineated subtype-specific metastatic pathways, showing distinct gene expression profiles associated with lung versus liver dissemination in HER2-positive and triple-negative breast cancers [39]. These findings support the integration of subtype-tailored molecular signatures into pre-surgical risk models.

Together, these molecular tools are promising and may eventually complement conventional criteria, pending prospective validation. They may allow a more personalized and biologically informed selection process for surgical candidates with oligometastatic breast cancer.

### 5.2. Artificial Intelligence and Digital Pathology

Artificial intelligence (AI) and computational pathology are rapidly transforming the landscape of oncologic diagnostics and surgical planning. In the context of metastatic breast cancer, these technologies offer tools to improve the precision of preoperative assessments, optimize patient selection for local therapies, and support intraoperative decision-making.

One area of rapid development is digital histopathology. Ivanov et al. demonstrated that AI algorithms applied to histological whole-slide images can accurately classify tumor grade and predict lymph node involvement, surpassing conventional manual assessment in reproducibility and speed [40]. Such applications may enhance pre-surgical risk stratification in the future, although current evidence is primarily derived from retrospective or proof-of-concept studies.

AI is also being applied to intraoperative and perioperative surgical decision support. Li et al. reviewed the expanding role of AI-assisted navigation in breast cancer surgery, highlighting innovations in image recognition, margin assessment, and real-time feedback systems [41]. These advances are further supported by the integration of augmented and mixed reality platforms, which enhance anatomical visualization and may improve resection outcomes in hepatic and pulmonary metastasectomy [42]. While current tools are largely confined to primary tumor surgery, their adaptation to thoracic and hepatic procedures could enable more accurate resections and reduce rates of incomplete (R1) excision. Moreover, predictive models developed through machine learning may help estimate individual recurrence risk and postoperative outcomes based on multidimensional clinical data. Tan et al. demonstrated the clinical feasibility of using AI-assisted 3D reconstruction of imaging and pathological features to guide resection planning in hepatic metastases [43]. This integrative approach improved intraoperative precision and reduced margin positivity in early-phase single-institution studies, and broader validation across diverse surgical settings is required. In parallel, Turner et al. have proposed an AI-based consensus algorithm to support resectability decisions in oligometastatic disease, integrating radiomic, clinical, and biological parameters [36]. These platforms offer scalable support for MDTs, particularly in resource-constrained or high-volume settings.

The promise of AI lies not only in its diagnostic utility but also in its integrative potential. AI systems capable of aggregating imaging, pathology, molecular, and clinical data could serve as decision-support platforms in multidisciplinary tumor boards, aiding in the selection of surgical vs. non-surgical interventions for oligometastatic disease although most applications remain investigational.

Although many of these applications are still under clinical validation, the convergence of AI and pathology marks a pivotal shift toward data-driven, personalized surgical oncology. From a medical and biological standpoint, the future potential of AI-driven platforms may extend beyond clinical decision support toward modeling the evolutionary trajectory of metastatic breast cancer. By integrating genomic alterations, transcriptomic signatures, radiomic phenotypes, systemic therapy response patterns, and longitudinal liquid biopsy dynamics, AI systems could eventually generate predictive models of clonal evolution and organ-specific metastatic fitness. Such biologically informed algorithms may allow clinicians to anticipate whether residual metastatic deposits represent stable, therapy-controlled clones amenable to local eradication, or emerging aggressive subclones with high systemic dissemination potential. In this scenario, surgical decision-making would no longer rely primarily on anatomical resectability or lesion count, but on probabilistic modeling of tumor behavior under continued systemic pressure. Although these applications remain theoretical and require rigorous prospective validation, they illustrate a paradigm shift toward evolution-informed surgical oncology, in which metastasectomy is guided by dynamic biological risk rather than static imaging criteria. As the complexity of metastatic breast cancer management increases, MDT decision-making has become central to optimizing patient outcomes. In this context, AI is emerging as a transformative tool capable of enhancing consistency, speed and individualization of clinical decisions, including those regarding surgery for oligometastatic disease.

Mooghal et al. emphasized the growing relevance of AI-supported platforms in the management of oligometastatic breast cancer, particularly in stratifying patients for locoregional interventions such as surgery or stereotactic ablative radiotherapy [44]. AI-driven tools can assist in evaluating large volumes of multimodal data—including imaging, histopathology, and genomic profiles—enabling more precise estimation of metastatic burden, treatment response, and recurrence risk. These capabilities may improve the timing and appropriateness of metastasectomy or other focal therapies.

Moreover, AI may play a role in standardizing MDT discussions, reducing inter-observer variability in interpretation and facilitating more equitable decision-making across centers. By integrating predictive modeling, AI can simulate treatment outcomes under different scenarios, providing valuable input for complex cases where evidence is limited or conflicting.

However, successful integration of AI into real-world MDT workflows faces several challenges. Mooghal et al. underscore the barriers related to data standardization, digital infrastructure, and the limited adoption of electronic health records in resource-constrained settings [44]. Additionally, regulatory and ethical frameworks for the clinical deployment of AI systems remain in development.

Despite these hurdles, the trajectory of AI in MDT planning is promising. If prospectively validated and operationalized within robust clinical frameworks, these technologies may have the potential to personalize surgical and locoregional treatment strategies, reduce decision-making time, and ultimately improve outcomes for patients with limited metastatic breast cancer.

## 6. Future Directions and Implementation Challenges

The integration of molecular diagnostics, AI, and digital pathology into surgical oncology presents a transformative opportunity to personalize care for patients with breast cancer metastases (Table 4). However, translating these innovations into clinical practice—particularly in the context of metastasis-directed surgery—requires careful attention to validation, scalability, and equity.

One major challenge is the need for prospective validation of AI tools and molecular assays in diverse, real-world settings. Many current models are trained on homogeneous populations or rely on retrospective data. Without external validation, their generalizability remains uncertain. As noted by Mooghal et al., the adoption of AI in oncology is particularly limited in low-resource settings due to insufficient digital infrastructure and inconsistent electronic health record (EHR) systems [44]. This disparity risks widening existing gaps in cancer care unless addressed through coordinated global efforts.

Standardization of data input formats, interoperability across platforms, and the development of regulatory frameworks for AI-driven clinical tools are additional priorities. Initiatives to benchmark performance metrics, such as predictive accuracy or clinical utility in decision-making, will be essential to support integration into multidisciplinary workflows.

Moreover, the convergence of multiple data streams—including imaging, genomic data, and liquid biopsy—poses logistical and analytical challenges. Ensuring that AI platforms can synthesize these data accurately and present actionable insights in real time is critical for their clinical relevance. As Garcia-Murillas et al. demonstrated, early detection of minimal residual disease through ctDNA monitoring could potentially guide the timing of surgical interventions, but such approaches must be operationalized within care pathways that are both timely and scalable [34].

Looking ahead, the focus will need to shift from proof-of-concept studies to clinical implementation, with attention to patient-centered outcomes, cost-effectiveness, and health system integration. Bridging these gaps will be key to fully realizing the promise of innovation in the surgical management of metastatic breast cancer.

### From Palliative to Precision: Rethinking Surgical Management in Visceral Breast Cancer Metastases

The surgical management of liver and lung metastases in breast cancer is increasingly moving beyond its traditional palliative role. Accumulating evidence suggests that, in selected patients, resection of visceral metastases has been associated with prolonged survival in selected retrospective series, particularly in the context of oligometastatic disease [18,20]. This shift is enabled by the refinement of systemic therapies, more nuanced biological profiling, and the availability of minimally invasive surgical and ablative techniques [22,30].

Multiple retrospective studies have reported encouraging outcomes, with 5-year OS rates of 30–60% following pulmonary or hepatic metastasectomy in carefully selected patients [20,21,30]. Long-term survival outcomes appear more favorable in patients with hormone receptor-positive or HER2-positive disease, limited metastatic burden, and long disease-free intervals. However, such data must be interpreted with caution. Most supporting evidence is retrospective, lacking uniform criteria for patient selection or control groups treated with systemic therapy alone [17,45]. In a recent retrospective series, a longer DFI—specifically beyond three years—was significantly associated with better progression-free survival following liver metastasectomy [17]. Prospective validation is urgently needed. In addition, Tinterri et al. reported a retrospective analysis of 61 patients with de novo metastatic breast cancer who underwent front-line chemotherapy followed by loco-regional treatment of the primary tumor [46]. After a median follow-up of 55 months, no statistically significant differences in PFS, DPFS, or OS were observed across subgroups defined by menopausal status, tumor subtype, number or site of metastases, or radiologic response to systemic therapy. Although a non-significant trend toward improved outcomes was noted in triple-positive tumors, no clearly identifiable subgroup derived a statistically significant survival advantage. While focused on primary tumor surgery rather than visceral metastasectomy, these findings further underscore the complexity of surgical decision-making in metastatic breast cancer and the difficulty in distinguishing prognostic selection from true therapeutic benefit.

Non-surgical alternatives, such as MWA and SBRT, have demonstrated high local control rates and favorable survival outcomes in inoperable patients or those with high surgical risk [28,31]. These modalities offer compelling advantages in terms of morbidity and recovery, but comparative trials are lacking, and the long-term oncologic equivalence to surgery remains uncertain. Importantly, direct comparisons between surgical resection, thermal ablation, and SBRT are substantially limited by heterogeneity across published studies. Available series differ markedly in patient selection criteria, including the number and size of metastases, the presence or control of extra-visceral disease, performance status, and prior lines of systemic therapy. Furthermore, molecular subtype distribution varies significantly between cohorts, and many studies span different systemic therapy eras, introducing confounding related to evolving endocrine, HER2-targeted, and cytotoxic regimens. Outcome definitions are also inconsistent, with variable reporting of overall survival, disease-free survival, progression-free survival, and local control. Differences in technical expertise, institutional volume, and treatment intent (curative vs. consolidative vs. palliative) further complicate interpretation. Consequently, the apparent similarities or differences in survival across modalities should not be interpreted as evidence of equivalence or superiority. Rather, they reflect the complexity of a biologically heterogeneous disease treated within diverse clinical contexts.

The growing integration of molecular diagnostics and AI offers further opportunity to refine decision-making. Circulating tumor DNA (ctDNA) assays have shown promise in detecting minimal residual disease and predicting relapse months before clinical or radiologic evidence emerges, enabling timely identification of patients who might benefit from metastasis-directed interventions [34]. Similarly, transcriptomic profiling may identify patients with favorable metastatic biology, while AI-based histopathology and imaging tools are being developed to assist with risk stratification and preoperative planning [38,40,41]. Tools, such as ctDNA and subtype-specific gene expression signatures, may, in the future, help refine surgical decision-making beyond purely anatomical criteria toward a biologically driven framework. Indeed, non-invasive prognostic biomarkers derived from liquid biopsy—such as CTCs, ctDNA, and circulating RNA-based signatures—may further refine patient stratification. CTC burden has long been associated with adverse outcomes in metastatic breast cancer, while dynamic changes in ctDNA levels may reflect systemic disease activity and minimal residual disease. Although these biomarkers are not currently used as definitive criteria for metastasectomy, their integration into multidisciplinary evaluation could, in the future, assist in distinguishing biologically indolent oligometastatic disease from early disseminated progression. At present, however, their role in guiding surgical decision-making remains investigational and requires prospective validation.

The central question is no longer “Can we operate?” but rather “Should we, and when?” Surgical feasibility alone is insufficient justification in an era increasingly guided by precision oncology. Instead, surgery must be offered within a multidisciplinary context, tailored to individual patient biology, disease kinetics, and therapeutic goals (Table 5). AI-enabled tools that integrate radiologic, pathologic, and genomic data are now emerging as adjuncts to MDT decision-making, offering scalable solutions for complex case triage and surgical planning [36,43,44].

Looking forward, the field must move from anecdotal experience to structured evidence. Prospective trials, real-world registries, and standardized criteria for surgical candidacy are essential. Equally important is the equitable implementation of emerging technologies to ensure access across diverse clinical settings. However, at present most molecular and AI-driven tools discussed herein should be regarded as adjunctive research instruments rather than standard-of-care decision-making platforms in metastasis-directed therapy.

Metastasectomy in breast cancer should be seen not as a technical endpoint, but as a strategic component of a personalized, biologically informed treatment paradigm. The next frontier will be prospective validation: defining how, when, and for whom surgical intervention meaningfully alters the natural history of metastatic breast cancer.

To synthesize the available evidence and facilitate clinical interpretation, Table 6 summarizes the principal local treatment modalities currently employed for liver and lung metastases from breast cancer, highlighting indications, candidate profiles, advantages, limitations, and level of evidence.

## 7. Conclusions

The surgical management of liver and lung metastases in breast cancer is progressively evolving from a historically palliative intervention toward a carefully individualized component of multimodal therapy. Retrospective series and registry-based analyses consistently report encouraging long-term survival outcomes following hepatic or pulmonary metastasectomy in highly selected patients, particularly those with limited metastatic burden, favorable tumor biology, prolonged disease-free intervals, and responsiveness to systemic treatment.

However, these findings must be interpreted with caution. The available evidence is predominantly retrospective and inherently subject to selection bias, immortal time bias, and confounding by tumor biology and treatment era. Patients selected for surgery often represent a biologically favorable subgroup with better performance status and access to contemporary systemic therapies, including endocrine agents, HER2-targeted treatments, CDK4/6 inhibitors, and antibody–drug conjugates. As a result, improved survival observed in surgical cohorts cannot be unequivocally attributed to metastasectomy itself. In the absence of adequately powered randomized trials directly comparing surgery plus systemic therapy versus systemic therapy alone in clearly defined oligometastatic populations, a causal survival benefit remains unproven.

Non-surgical local therapies, including thermal ablation and stereotactic body radiotherapy, further expand the therapeutic landscape, offering effective local control in selected patients. However, meaningful cross-modality comparisons between surgery, thermal ablation, and SBRT remain inherently limited by substantial heterogeneity across studies. Variations in patient selection, metastatic burden, molecular subtype distribution, systemic therapy exposure, timing of local intervention, institutional expertise, and endpoint definitions preclude robust comparative conclusions. As a result, currently available data do not allow definitive statements regarding the superiority or equivalence of any single modality. Prospective, biologically stratified trials with standardized outcome measures are essential to clarify the relative contribution of each approach within multimodal care.

Emerging molecular tools such as circulating tumor DNA and transcriptomic profiling, together with artificial intelligence–assisted imaging and decision-support systems, hold promise for refining patient selection and integrating biological risk stratification into surgical planning. Nevertheless, most of these technologies remain investigational in the specific context of metastasis-directed surgical decision-making and require rigorous prospective validation before clinical adoption.

Ultimately, metastasis-directed surgery in breast cancer should not be viewed as a universally survival-enhancing intervention, but rather as a potential component of a personalized, multidisciplinary treatment strategy in rigorously selected patients. The future of this field lies in prospective validation, standardized biologically informed selection criteria, and integration within structured precision oncology frameworks.

## Figures and Tables

**Table 1:** Summary of outcomes after liver resection for breast cancer liver metastases.

Study/Series	Design	Patients (*n*)	Median OS (Months)	5-Year OS (%)	Key Predictors of Outcome
LIBREAST [20]	Multicenter registry	~200	~60	60%	HR+/HER2+, R0 resection, response to systemic therapy
Masuda et al. [21]	Retrospective	35	49	46%	Disease-free interval >12 months, limited liver disease
Rahnemai et al. [18]	SEER-based nomogram	122	38	39%	Single metastasis, ER+/HER2+

**Table 2:** Comparative outcomes of locoregional therapies for BCLM.

Treatment Modality	Median OS	5-Year OS (%)	Patients (*n*)	Notes
Microwave ablation	5.6 years	89%	17	Highly selected group
LITT	2.2 years	22%	491	Earlier era cohort
LITT + TACE	2.1 years	15%	370	Combined therapy
TACE alone	0.8 years	4%	242	Advanced disease, palliative intent

LITT = laser interstitial thermal therapy; OS = overall survival; TACE = transarterial chemoembolization. Data source: Vogl et al. [22].

**Table 3:** Summary of outcomes and selection criteria in pulmonary metastases from breast cancer.

Study/Source	Treatment Modality	Patients (*n*)	Median OS	5-Year OS (%)	Key Selection Criteria	Notable Findings
Handy 2019 [30]	Pulmonary metastasectomy	157	Not specified	38% (LN+) vs. 69% (LN-) at 3 years	≤3 lesions, R0 feasible, no uncontrolled extra-thoracic disease, disease-free interval > 12–24 months	LN involvement significantly worsens prognosis; repeat PM feasible in selected cases
Franceschini 2024 [31]	SBRT (lung and liver OMBC)	24 (lung subgroup)	16.5 months	51.9% at 3 years	Oligometastatic disease, controlled systemic burden	1-, 2-, 3-year local control: 94.9%, 91%, 87.5%; well tolerated
Meng 2021 [28]	Microwave ablation	32	36 months	17.8% at 5 years	≤3 lung lesions, no progressive extra-pulmonary disease	96.9% 1-year OS; primary efficacy rate 97.8%; low complication profile

**Table 4:** Emerging technologies in surgical decision-making for visceral breast cancer metastases.

Innovation	Application	Impact
ctDNA [34]	MRD detection, relapse prediction	Identifies candidates for early intervention
Transcriptomic profiling [38]	Prediction of organ-specific metastases	Supports personalized surveillance and resection strategies
Subtype-specific signatures [39]	Subtype-based metastatic pattern identification	Refines patient selection by subtype biology
AI Histopathology [40]	Lymph node status, tumor grading	Enhances surgical risk stratification
AI-assisted surgical navigation [41]	Margin detection, intraoperative guidance	Improves resection accuracy and reduces margin positivity
3D AI reconstruction for liver resection [43]	Pre-surgical planning in hepatic metastases	Enhances intraoperative planning and precision
AI resectability consensus algorithm [36]	Multimodal decision-making in oligometastatic disease	Improves selection in complex or low-resource cases
AI-supported MDT platforms [44]	Scenario modeling and MDT decision support	Standardizes MDT decisions and optimizes timing

**Table 5:** Criteria for patient selection for surgical management of visceral metastases.

Criterion	Liver Metastases	Lung Metastases
Number of lesions	≤3–5	≤3
Disease-free interval	>12 months preferred	>12–24 months
Extra-visceral disease	Controlled or absent	Controlled or absent
Systemic disease response	Favorable	Favorable
Tumor biology	HR+/HER2+ preferred	HR+/HER2+ preferred
Performance status	ECOG 0–1	ECOG 0–1
Resection feasibility	R0 achievable	R0 achievable
Comorbidities	Acceptable for surgery	Pulmonary reserve adequate

**Table 6:** Comparative Overview of Local Treatment Modalities for Liver and Lung Metastases in Breast Cancer.

Modality	Typical Indications	Ideal Candidate Profile	Advantages	Limitations	Level of Evidence
Hepatic Metastasectomy	Limited liver metastases (commonly ≤3–5 lesions); feasibility of R0 resection; controlled or absent extrahepatic disease	HR+/HER2+ subtype; good performance status; long disease-free interval; favorable response to systemic therapy	Potential for prolonged survival in selected patients; complete macroscopic disease clearance; repeatable in selected cases	Invasive; perioperative morbidity; strong selection bias; absence of randomized trials	Retrospective cohort studies; registry analyses
Pulmonary Metastasectomy	Isolated or oligometastatic lung nodules; no uncontrolled systemic progression	Limited number of nodules; controlled systemic disease; DFI >12–24 months	Low perioperative mortality (especially VATS); repeatable; pathological confirmation	No randomized controlled trials; heterogeneity in patient selection; unclear survival attribution	Retrospective institutional series
Microwave Ablation (MWA)/Radiofrequency Ablation (RFA)	Small lesions (typically <3 cm); unresectable disease; high surgical risk	Limited tumor burden; lesions in accessible anatomical locations	Minimally invasive; repeatable; shorter hospital stay	Limited comparative survival data; local recurrence risk in larger lesions; operator-dependent outcomes	Small retrospective series; limited prospective data
Stereotactic Body Radiotherapy (SBRT)	Oligometastatic or oligoprogressive disease; inoperable patients; consolidative therapy	Controlled systemic disease; limited number of metastases	Non-invasive; high local control rates; well tolerated	Lack of definitive OS benefit in randomized trials (e.g., NRG-BR002 [32]); heterogeneous protocols	Phase II trials; one negative phase II RCT; retrospective studies
Combined Multimodal Approach (Systemic + Local Therapy)	Oligometastatic disease responding to systemic therapy	Favorable tumor biology; durable systemic control	Integrates systemic disease control with local eradication; individualized strategy	Evidence largely observational; optimal sequencing undefined	Retrospective data; emerging prospective trials

DFI: Disease-free interval; HR: Hormone receptor; HER2: Human epidermal growth factor receptor 2; VATS: Video-assisted thoracoscopic surgery; OS: Overall survival; RCT: Randomized controlled trial.

## Data Availability

No new data were created or analyzed in this study.
